# Identification of the miRNA targetome in hippocampal neurons using RIP-seq

**DOI:** 10.1038/srep12609

**Published:** 2015-07-28

**Authors:** Josephine Malmevik, Rebecca Petri, Thies Klussendorf, Pina Knauff, Malin Åkerblom, Jenny Johansson, Shamit Soneji, Johan Jakobsson

**Affiliations:** 1 Laboratory of Molecular Neurogenetics, Department of Experimental Medical Science, Wallenberg Neuroscience Center and Lund Stem Cell Center, Lund University, SE-221 84 Lund, Sweden

## Abstract

MicroRNAs (miRNAs) are key players in the regulation of neuronal processes by targeting a large network of target messenger RNAs (mRNAs). However, the identity and function of mRNAs targeted by miRNAs in specific cells of the brain are largely unknown. Here, we established an adeno-associated viral vector (AAV)-based neuron-specific Argonaute2:GFP-RNA immunoprecipitation followed by high-throughput sequencing to analyse the regulatory role of miRNAs in mouse hippocampal neurons. Using this approach, we identified more than two thousand miRNA targets in hippocampal neurons, regulating essential neuronal features such as cell signalling, transcription and axon guidance. Furthermore, we found that stable inhibition of the highly expressed miR-124 and miR-125 in hippocampal neurons led to significant but distinct changes in the AGO2 binding of target mRNAs, resulting in subsequent upregulation of numerous miRNA target genes. These findings greatly enhance our understanding of the miRNA targetome in hippocampal neurons.

MicroRNAs (miRNAs) are small, non-coding RNAs that bind to complementary messenger RNA (mRNA) targets, thereby causing a decrease in target mRNA activity. Increasing evidence indicates an important role for miRNAs in the brain, where multiple miRNAs are highly expressed and many are enriched in specific brain regions^1^. Functional studies suggest that miRNAs regulate a number of cellular processes relevant for neuronal functions, including synaptic plasticity, dendritic branching, adult neurogenesis and neuronal survival^1^,^2^. In the hippocampus, depletion of the entire miRNA pathway using conditional deletion of *Dicer*, results in an improved performance in cognitive tests, indicating that miRNAs are key molecules in controlling learning and memory^3^. Deletion of *Dicer* ultimately results in neuronal cell death, implicating a role for miRNAs in neurodegeneration^3^,^4^. The miRNome of hippocampal neurons, which consists of several hundred miRNAs, has been extensively studied and important roles have been attributed to individual miRNA families, such as miR-34 and miR-124^5^,^6^.

Although we now have substantial knowledge about the expression pattern of different miRNAs in the brain, it is paradoxical that we know very little about which mRNAs that are targets of miRNAs in different neuronal cell types. It has been speculated that miRNAs regulate large networks of genes since each miRNA can target several hundred transcripts and each transcript can be regulated by several miRNAs^7^. However, the identification and functional analysis of genes targeted by miRNAs in specific subsets of neurons is challenging, and therefore the identity of the network of miRNA target genes in the brain remains largely unknown.

The function of miRNAs is thought to be primarily restricted to the RNA-induced silencing complex (RISC), which consists of several factors including argonaute (AGO) proteins. In the RISC, miRNAs bind to mRNAs and inhibit their translation or elicit their degradation^7^. Biochemical isolation of AGO proteins has recently been employed to analyse bound miRNAs and their target genes^8^,^9^,^10^,^11^. The combination of AGO protein isolation with RNAse digestion and next generation sequencing as used in techniques such as HITS-CLIP and PAR-CLIP allows for a snapshot of miRNA-mRNA interactions^8^,^11^. However, these techniques are difficult and ultimately depend on small RNA-seq of a limited amount of material, which potentially causes sequencing bias^12^. These drawbacks complicate quantitative assessments of e.g. the relative enrichment of different transcripts in the RISC. To circumvent these issues, we have here established a novel approach allowing neuron-specific RNA immunoprecipitation followed by next generation sequencing (RIP-seq) and investigated miRNA targets in hippocampal neurons at a genome-wide level.

## Results

### Establishment of neuron-specific RIP in the hippocampus of the mouse brain

**Figure 1:**
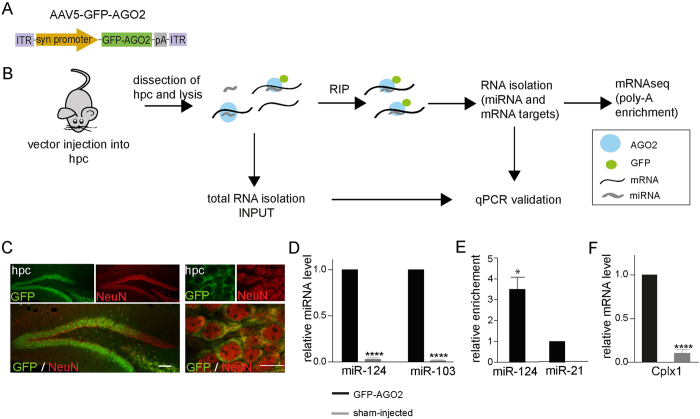
Establishment of neuron-specific RIP in the mouse brain (**A**) Diagram of the AAV5-GFP-AGO2 vector. (**B**) Diagram of the procedure of RNA-interacting protein immunoprecipitation (RIP-seq) (**C**) The expression of GFP-AGO2 is shown in low (left panel) and high (right panel) magnification. Scale bars 100 μm (left panel) and 10 μm (right panel). hpc- hippocampus (**D–F**) RNA from the hippocampus of was analysed using qPCR after RIP. Data are represented as mean ± SEM. (**D**) miRNAs such as miR-124 and miR-103 were detected only in RIP samples where GFP-AGO2 was expressed (****p < 0.0001; unpaired t-tests; n = 4 of each). (**E**) miR-124 was more than three-fold higher detected in comparison to the glia-enriched miR-21 in RIP samples, when compared to INPUT fractions (*p < 0.05; unpaired t-test; n = 4). (**F**) The highly expressed known miRNA target, *Cplx1*, was enriched in GFP-AGO2-injected brains compared to sham-injected brains (****p < 0.0001; unpaired t-test; n = 4).

To investigate miRNA targets in hippocampal neurons at a transcriptome level, we optimised a neuron-specific GFP-AGO2 RIP approach allowing for the isolation of miRNAs and mRNAs from the mouse brain without the need of crosslinking (see Experimental Procedures for details), thereby taking advantage of the fact that the AGO2 protein is in direct contact with miRNAs and mRNAs in the RISC. We generated adeno-associated viral serotype 5 (AAV5) vectors expressing a GFP-AGO2 fusion protein (AAV5-GFP-AGO2) under the control of a synapsin promoter (Fig. 1A). The vector design allows for RIP of miRNA and mRNA bound to the GFP-AGO2 fusion protein by using a GFP antibody (Fig. 1B). Moreover, neuron-specific RIP is ensured by the synapsin-driven expression of the fusion protein. We targeted the mouse hippocampus by performing bilateral three-site injections resulting in GFP-AGO2 expression in the cytoplasm of hippocampal neurons in the dentate gyrus, CA1 and CA3 (Fig. 1C; left panel). The GFP expression resembled endogenous cytoplasmic AGO2 localisation (Fig. 1C; right panel). To validate our RIP-approach, hippocampal tissue was extracted eight weeks after injection, followed by RIP and RNA extraction. Using qRT-PCR, we found a high enrichment of miR-103 and miR-124 in AAV5-GFP-AGO2-injected animals compared to sham-injected animals, confirming efficient RIP of miRNAs (Fig. 1D). Moreover, the neuron-specific expression of the GFP-AGO2 fusion protein was confirmed by analysing the ratio of the neuron-specific miR-124 to the glia-enriched miR-21^13^,^14^,^15^ in RIP fractions compared to INPUT fractions. We found miR-124 to be more than three-fold enriched compared to miR-21 in RIP samples (Fig. 1E). We also found a specific enrichment of the previously identified miRNA target gene *Cplx1*^8^ in AAV5-GFP-AGO2 injected animals compared to sham-injected animals (Fig. 1F), thereby confirming efficient RIP of miRNA-targeted mRNAs. Thus, our experimental approach allows for RIP of miRNAs and target mRNAs bound to the GFP-AGO2 fusion protein specifically in hippocampal neurons.

### Analysis of AGO2 binding at a transcriptome level

**Figure 2:**
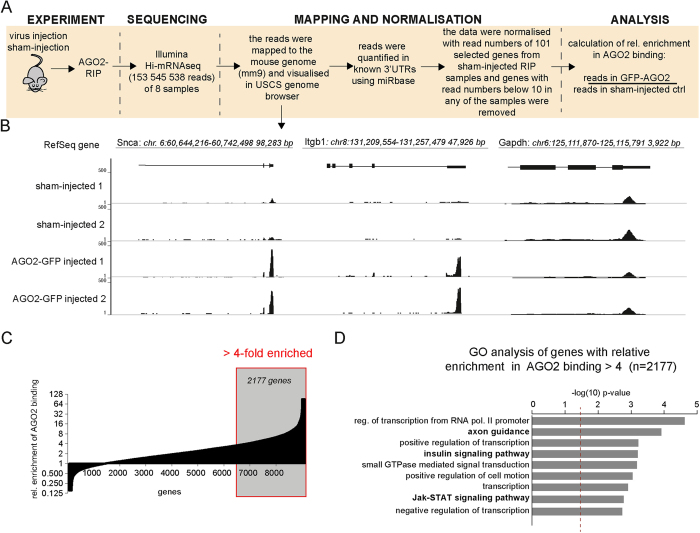
Analysis of mRNA composition in RISC (**A**) Reads obtained from the RISC using high-throughput next generation sequencing were mapped to the mouse genome and quantified. (**B**) Abundant reads were mapped to the 3′-untranslated regions (3′UTRs) of α-synuclein (Snca; left panel) and integrin β 1 (Itgb1; middle panel). The 3′UTR of glyceraldehyde 3-phosphate dehydrogenase (GAPDH) only had negligible reads in RIP-seq samples (right panel). (**C**) Genes detected by RIP-seq were plotted against their relative enrichment in AGO2 binding, showing a progressive increase (see Supplementary Table S1). A 4-fold enrichment in RIP samples (grey box) was chosen as cut-off for identifying AGO2-bound mRNAs. (**D**) GO analysis of genes that were enriched more than 4-fold in the RISC.

In order to analyse miRNA targets at a genome-wide level, we generated polyA-selected cDNA libraries from RIP samples of animals injected with AAV5-GFP-AGO2 and sham controls. We performed high-throughput next generation sequencing on these samples and the resulting reads were subsequently mapped to the mouse genome. For the bioinformatics analysis, we focused on reads present in the 3′UTRs to avoid bias due to potential RNA degradation occurring during the RIP procedure (Fig. 2A). We found abundant reads in the 3′UTRs in RIP-seq samples, but not in sham control samples, of many well-characterised miRNA target genes, such as α-synuclein (Snca) and integrin β1 (Itgb1;Fig. 2B, left and middle panel). On the other hand, genes without any conserved sites for miRNAs within vertebrates, such as GAPDH (TargetScanMouse 6.2,^16^), displayed negligible reads in RIP-seq samples, similar to the sham controls (Fig. 2B, right panel), thereby confirming a high specificity and low background of our optimised RIP technique. We confirmed the robustness of our approach in an independent biological replicate experiment.

To assess which genes were most enriched in the RISC, we next calculated the relative enrichment of AGO2 binding by dividing the number of reads in RIP-seq samples by that of sham control samples in all known 3′UTRs of the mouse genome. The AGO2-mRNA binding displayed a progressively increased enrichment, which is likely to reflect differences in miRNA-mRNA base pairing as well as the presence of several miRNA target sites on mRNAs (Fig. 2C). In order to delineate mRNAs that are targets of miRNAs, we used a cut-off at 4-fold enrichment in RIP samples over control samples, leading to the identification of 2177 genes that are bound to AGO2 in hippocampal neurons. These genes are thus likely to be miRNA targets in this cell population (Fig. 2C, grey box). To reveal biological processes regulated by miRNAs in hippocampal neurons, we performed gene ontology analysis of the 2177 target genes. We found that miRNAs regulate multiple large gene networks involved in essential cellular functions such as cell signalling, transcription and axon guidance (Fig. 2D).

To confirm the specificity of our approach, we first investigated whether relative enrichment in AGO2 binding correlates to the presence of miRNA binding sites in the 3′UTR. To this end, we plotted genes according to increasing AGO2 binding vs. the cumulative presence of predicted, conserved miRNA target sites (TargetScan^16^) for five different miRNAs. Predicted target genes of three miRNAs (miR-124, miR-125 and let-7) known to be expressed in hippocampal neurons, were found to be highly enriched in the fraction of AGO2-bound mRNAs (Supplementary Fig. S1A, three left-most graphs). In these cumulative graphs, most genes are present at the right-most end of the graph representing high relative enrichment in AGO2 binding, thus being subjected to miRNA-mediated targeting. On the other hand, cumulative graphs for mRNAs with target sites for the glia-enriched miR-21 and the non-neural-expressed miR-292, did not display any obvious enrichment for AGO2-binding (Supplementary Fig. S1A, two right-most graphs). We also confirmed that genes expressed in glial cells^17^ were not enriched in AGO2-bound transcripts while neuron-expressed transcripts were (Supplementary Fig. S1B-C). Taken together, this analysis validates our RIP-seq approach by linking enrichment in AGO2 binding to the presence of conserved, predicted target sites for miRNAs expressed in neurons and confirms that AGO2-bound transcript are genes expressed in neurons but not glia.

### RIP analysis reveals mode-of-action of sponge vectors

**Figure 3:**
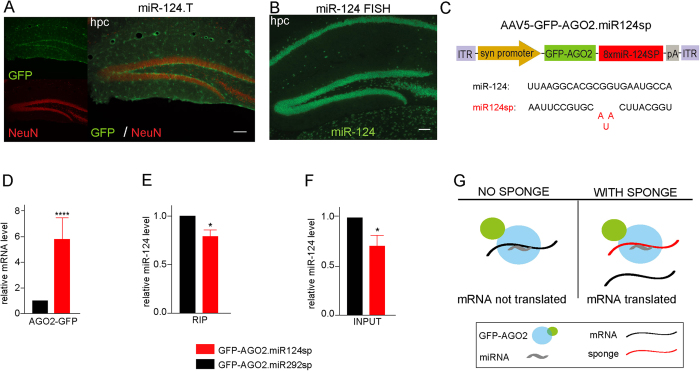
Changes in RISC composition after miRNA inhibition (**A**) The generation of GFP miRNA sensor mice for miR-124 allows for the visualisation of miRNA activity at a single-cell resolution. In this setup, GFP is produced in cells where there is no activity of the miRNA of interest. In this figure, GFP expression is not detected in hippocampal neurons (NeuN; red) of GFP miR-124 sensor mice, as miR-124 activity degrades the GFP transcript. Scale bar 100 μm. (**B**) Expression of miR-124 is also seen seen fluorescent *in situ* hybridisation in neurons of the entire adult mouse hippocampus. Scale bar 100 μm. (**C**) Diagram of the AAV5-GFP-AGO2.miR124sp vector. (**D**) The GFP-AGO2.miR124sp sequence was six-fold higher enriched in RIP samples of miR124sp-injected brains, than in GFP-AGO2.292sp injected controls (****p < 0.0001; unpaired t-test). The values were normalised to the levels of the known miRNA target *Cplx1*. (**E**) miR-124 was detected at slightly lower levels in the RIP samples after inhibition using miR124sp in comparison to GFP-AGO2.292sp-injected controls (*p < 0.05; unpaired t-test; n = 5). (**F**) A lower level of total miR-124 was also detected in the INPUT samples (total RNA; *p < 0.05; unpaired t-test; n = 5). (**G**) Schematic of the function of miR-sponges. Data are represented as mean ± SEM.

A key aspect of miRNA biology that remains poorly understood is how individual miRNA families contribute to mRNA silencing. miR-124 is an evolutionarily conserved miRNA that is highly expressed in the brain^18^. The expression of miR-124 is initiated in neural progenitors and reaches high levels in mature neurons^13^,^19^. Moreover, several studies have demonstrated a role for miR-124 in promoting neuronal differentiation^13^,^19^,^20^,^21^. miR-124 is highly expressed in hippocampal neurons (Fig. 3A,B) and several studies link miR-124 to hippocampal functions, however, the underlying molecular mechanisms remain largely unresolved^5^,^13^.

To analyse the contribution of miR-124 to miRNA regulation in hippocampal neurons, we generated AAV5 vectors that express a miR-sponge for miR-124 in combination with a GFP-AGO2 fusion protein (AAV5-GFP-AGO2.miR124sp, Fig. 3C). miR-sponges are imperfectly complementary transgenic transcripts with multiple bulged miRNA-binding sites that result in efficient inhibition of miRNAs *in vivo*^13^,^22^,^23^,^24^,^25^,^26^. In this experiment, the GFP-AGO2 fusion protein was expressed together with the sponge sequence as one single transcript under the control of the synapsin promoter to allow for neuron-specific RIP from the RISC only in cells where miR124sp is expressed (Supplementary Fig. S2). As a negative control, we also performed RIP on brains injected with a sponge vector for miR-292, which is a miRNA absent from the brain.

To investigate whether the miR-124 sponge transcript was incorporated into the RISC, we performed qRT-PCR analysis on RIP samples using a primer set recognising the vector-derived sponge transcript. Using qRT-PCR analysis we found that the transgenic GFP-AGO2.miR124sp transcript was efficiently incorporated into the RISC in contrast to that of the GFP-AGO2.miR292sp control (Fig. 3D). We also found that mature miR-124 levels in the RISC were only slightly decreased by the expression of AAV5-GFP-AGO2.miR124sp (Fig. 3E). A similar alteration of miR-124 level was detected in the INPUT fractions, which corresponds to all mature miR-124 in the hippocampus (Fig. 3F). Taken together, these data suggest a model for sponge-function in which miR-sponge vectors do not act by preventing the targeted mature miRNA to be loaded into the RISC, but rather occupies the RISC at the site that would otherwise be destined for the mRNA of miR-124 target genes, resulting in their release and de-repression (Fig. 3G).

### Inhibition of miR-124 alters the miRNA-targetome

**Figure 4:**
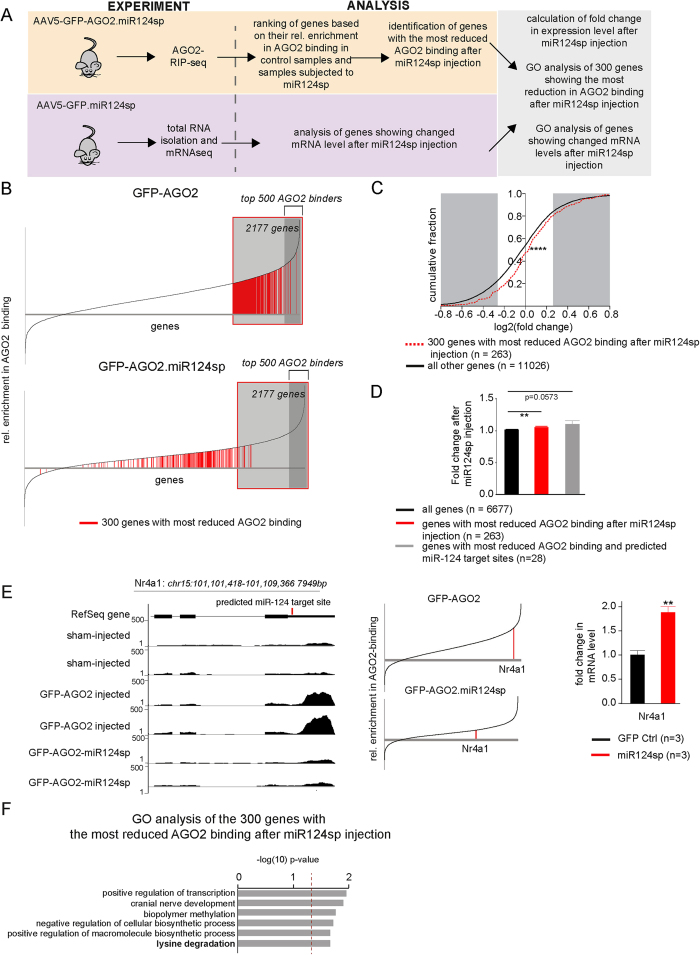
miR-124 inhibition causes prominent changes in AGO2 binding and de-repression of mRNAs (**A**) Schematic of experimental setup and conducted analyses. (**B**) Relative enrichment in AGO2 binding for each mRNA detected by RIP-seq in GFP-AGO2 injected mice (upper panel) and in mice injected with GFP-AGO2.miR124sp (lower panel) was plotted cumulatively. The top 300 genes showing the greatest decrease in relative AGO2 binding following GFP-AGO2.miR124sp injection, are depicted in red bars (see Supplementary Table S2). (**C**) When analysing the transcriptome using mRNAseq, we detected low-level, but significant changes in mRNA levels after miR-124 inhibition. The fold change (as log2 values) was plotted cumulatively for each gene representing the transcripts with the largest change in AGO2 binding after miR124sp injection. Genes showing the greatest reduction in relative AGO2 binding after miR-124 inhibition (red, dotted graph, n = 263)) were significantly different from that of all other genes (black graph; ****p < 0.0001; Kolmogorov-Smirnov Z test, n = 11026). (**D**) The fold change in mRNA expression level was significantly higher for the genes with the greatest decrease in AGO2 binding after miR124sp injection (red bar; **p < 0.01; unpaired t-test), and close to significant for the genes with the greatest decrease in AGO2 binding that were also predicted miR-124 target sites (TargetScan; grey bar), in comparison to that of all genes detected (black bar; n = 6677). Data are represented as mean ± SEM. (**E**) Example demonstrating the validated miR-124 target gene Nr4a1. Abundant reads were mapped to the 3′UTR of Nr4a1 in RIP-seq samples (left panel). In GFP-AGO2.miR124sp-treated samples, only negligible reads were detected, showing the release of Nr4a1 from the RISC after miR124sp treatment. In line with this, Nr4a1 demonstrated a prominent reduction in AGO2 binding (middle panel) and an increased mRNA expression after miR-124 inhibition (right panel; mean ± SEM; **p < 0.01; unpaired t-test). (**F**) GO of the 300 genes showing the greatest decrease in relative AGO2 binding (p < 0.05; F).

In order to investigate the composition of RISC-bound mRNAs after inhibiting miR-124, we performed RIP-seq experiments on hippocampal neurons expressing AAV5.GFP.miR124sp, (without exogenous AGO2 expression used for RIP, Fig. 4A). In line with the fact that miR-124 is highly expressed in hippocampal neurons, we found that miR-124 inhibition led to major changes in the mRNA-composition of the RISC. When miR124sp was expressed in hippocampal neurons, the mRNAs from 494 out of the 2177 genes, previously identified as miRNA-targets, were lost from the RISC.

We selected the 300 miRNA target genes displaying the greatest decrease in relative enrichment of AGO2 binding following miR-124 inhibition (Fig. 4B; red bars) and investigated whether loss of AGO2 binding correlated with changes in gene expression levels. We found that reduced enrichment in AGO2 binding following miR-124 inhibition resulted in increased mRNA expression level as monitored by mRNA-seq (Fig. 4C, ****p < 0.0001 Kolmogorov-Smirnov Z test). In accordance with several previous studies^27^,^28^,^29^, the magnitude of increased expression of the 300 miR-124 target genes following sponge expression was modest but highly significant (fold change 1.06+/− 0.015 SEM, Fig. 4D). To further characterise how direct miR-124 targets are affected by miR-124 inhibition, we selected genes with reduced enrichment in AGO2 binding that also have conserved, predicted miR-124 target sites (TargetScan). This provided us with 28 genes of which some have been previously experimentally validated as miR-124 targets, including nuclear receptor subfamily 4, group A, member 1 (Nr4a1), which displayed both reduced AGO2 binding and increased mRNA levels after miR-124 inhibition (Fig. 4E). Together, these 28 genes showed a slightly higher degree of increased mRNA levels after miR-124 inhibition when compared to the top 300 genes showing the most decrease in relative enrichment of AGO2 binding, although this difference did not reach significance (Fig. 4D; fold change 1.1+/− 0.05 SEM, p = 0.0573). In summary, these data show that decreased AGO2 binding following inhibition of miR-124 is associated with a modest but significant increase in mRNA levels.

We then set out to identify the function of the genetic network controlled by miR-124 in hippocampal neurons. We performed gene ontology and network analysis on the top 300 genes displaying the greatest decrease in AGO2 binding following inhibition of miR-124. Genes with reduced AGO2 binding after inhibition of miR-124 were enriched for functions related to transcription, nerve development and metabolic processes (Fig. 4F).

### Inhibition of miR-125 reveals distinct targets but similar dynamics of miRNA regulation compared to miR-124

In order to confirm that our results can be applied to miRNAs with broader expression profiles, we also investigated how inhibition of miR-125 affects AGO2 binding in hippocampal neurons. miR-125 is a brain-enriched miRNA family, unrelated to miR-124, that is also highly expressed and active in hippocampal neurons but also present in other cells of the brain such as glia^30^. Several studies have demonstrated a role for miR-125 in the regulation of neuronal differentiation and synaptic function^30^,^31^,^32^,^33^.

**Figure 5:**
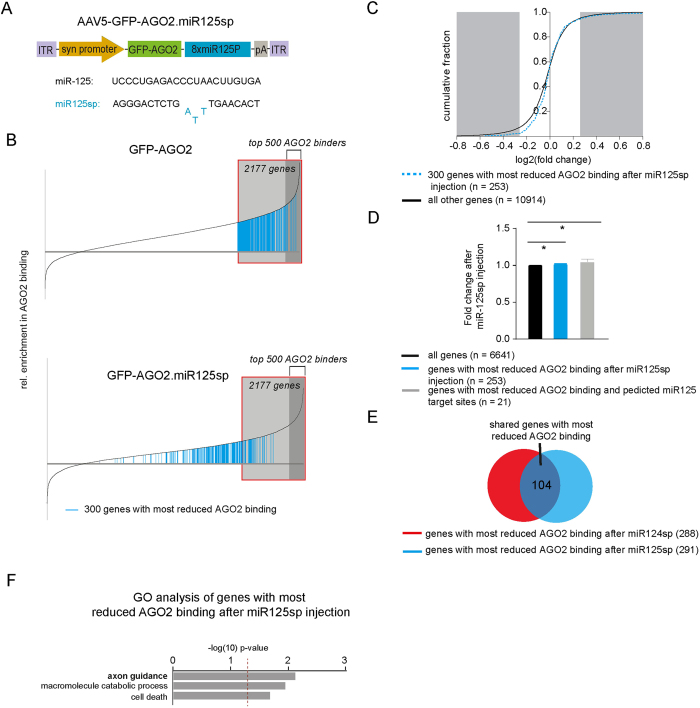
Changes in RISC composition and mRNA expression after miR-125 inhibition (**A**) Diagram of the AAV5-GFP-AGO2.miR125sp vector. (**B**) Relative enrichment in AGO2 binding for each mRNA detected by RIPseq in AGO-GFP control injected mice (upper panel) and in mice injected with GFP-AGO2.miR125sp (lower panel) was plotted cumulatively. The top 300 mRNA transcripts showing the greatest reduction in relative AGO2 binding following miR125sp injection, are depicted in blue bars (see Supplementary Table S3). (**C**) We detected changes in mRNA levels after miR-125 inhibition. The fold change (as log2 values) was plotted cumulatively for each gene with the greatest decrease in AGO2 binding after miR125sp injection (n = 253). Genes that showed the greatest reduction in relative AGO2 binding after miR-125 inhibition (blue, dotted graph) showed a trend of being less downregulated in comparison of that of all other genes (black graph), however this did not reach significance (p = 0.062; Kolmogorov-Smirnov Z test). (**D**) The fold change in mRNA expression level was significantly higher for the genes with reduced AGO2 binding after miR125sp injection (blue bar) and the most changed genes with predicted miR-125 target sites (TargetScan, grey bar) in comparison to that of all genes detected in RIP-seq (black bar; *p < 0.05; unpaired t-test n = 6641). Data are represented as mean ± SEM (**E**) Inhibition of miR-125 resulted in reduction in AGO2 binding in a different set of genes than that of miR-124. (**F**) GO of the 300 genes showing the greatest reduction in relative AGO2 binding (p < 0.05).

We inhibited miR-125 in hippocampal neurons by the same approach used for miR-124 and performed RIP-seq analysis (Fig. 5A). Similar to when miR-124 was inhibited, the inhibition of miR-125 also led to major changes in the mRNA composition of the RISC where mRNAs from 384 out of the 2177 AGO2-bound genes were lost from the RISC (Fig. 5B). We also found that reduction of AGO2 binding was correlated with significant but modestly increased mRNA levels when inhibiting miR-125 (Fig. 5C,D). However, the majority of the genes displaying reduced AGO2 binding upon miR-125 inhibition were different to when inhibiting miR-124 (Fig. 5E). In addition, GO analysis of genes with reduced AGO2 binding after inhibition of miR-124 and miR-125 respectively (Figs 4F), confirmed that these two miRNAs regulate separate sets of genes. GO analysis demonstrated that miR-125 controls axon guidance pathways, which is in line with previous observations on miR-125 and synaptic plasticity^30^,^32^, and also functions related to catabolic processes and cell death.

## Discussion

In order to understand the regulatory role of miRNAs in neurons of the brain, it is necessary to perform neuron-specific analysis of miRNAs and their target genes. While physical enrichment of certain cell types using for example FACS or laser capture microdissection offers a potential solution to this problem, these techniques suffer from several limitations since they are highly laborious, give low yield of material and are often associated with tissue manipulation that may alter gene expression. To avoid these issues, we have established a neuron-specific AGO2-RIP-seq approach, which captures miRNA targets during their biogenesis and functions within neurons *in situ*. In that way, the need for physical enrichment is circumvented and allows for the application of the technique on brain tissue, which is generally difficult to dissociate. Moreover, AGO2-RIP-seq avoids physical damage and cellular stress to living cells.

Our RIP-seq approach uses a GFP-AGO2 fusion protein, which allows for highly specific isolation of the RISC through the use of a GFP antibody that shows only very limited cross-reaction to other mouse proteins. Epitope-tagged AGO2 has been widely used to study RISC function and to immunopurify bound miRNAs and mRNAs. It has been shown that the fusion of the AGO2 protein to an epitope tag does not lead to any changes in AGO2 function^11^,^34^,^35^,^36^. By analysing the GFP intensity following AAV5-GFP-AGO2 injection, we detected a very low level of GFP-AGO2 protein expressed in hippocampal neurons. The fusion protein showed cytoplasmic localisation, without any aggregation, resembling endogenous AGO2 expression. This is in line with previous studies conducted in *Drosophila* and mouse, where the over-expression level of tagged AGO proteins appears to be limited by a negative feedback loop^34^,^37^. Thus, the use of AAV5-GFP-AGO2 is unlikely to affect the native miRNA profiles and their target mRNAs.

A key aspect of the RIP-seq methodology is that it allows for quantitative assessment of the relative enrichment in AGO2 binding due to the use of poly-A selected cDNA libraries. By taking advantage of this feature, we found that mRNA targets display a progressively increased enrichment in AGO2 binding, which most likely reflects differences in target site complementarity between mRNA-miRNA target sites and the fact that some mRNAs are regulated by several miRNAs simultaneously. We also used AGO2-RIP-seq to provide an insight into the genetic network regulated by miRNAs in hippocampal neurons. We identified more than two thousand miRNA target genes in the RISC, confirming that miRNAs regulate large networks in neurons. Functional annotation of these genes demonstrated that miRNAs control fundamental processes that are vital for hippocampal neurons, including cell signalling and transcription. Taking these facts into consideration, dysregulation or disruption of the miRNA network in hippocampal neurons is likely to result in severe consequences. This is in agreement with previous *Dicer* knockout studies, where the depletion of the entire miRNA pathway ultimately led to severe neurodegenerative phenotypes^3^,^4^.

A key question in the miRNA field is how individual miRNA families contribute to the regulation of neuronal transcripts. To probe this issue, we inhibited miR-124 and miR-125 using so-called AAV-sponge vectors. Several observations suggest that the AAV-sponges efficiently inhibit their target miRNAs. First, the vector contains a GFP-AGO2 fusion protein on the same transcript as the sponge. With this setup GFP is only detectably expressed when the level of sponge transcripts reaches saturation resulting in the inhibition of the target miRNA^23^. We found high-level GFP-expression after injection of AAV-sponge vectors. We have also previously demonstrated that the miR-124 and miR-125 sponge sequences used in the current study efficiently de-repress target transcripts using luciferase assays^13^,^30^. Finally, our RIP-qRT-PCR results suggest a model where miR-sponges inhibit their target miRNA by being incorporated into the RISC where they act in a manner that resembles dominant-negative molecules. This suggests that miR-sponges may have limited off-target effects, as they do not cause a major alteration of the population of mature miRNAs in the cell or in the RISC.

We found that inhibition of miR-124, which is highly and specifically expressed in neurons^13^, led to significant changes in the composition of mRNAs bound to the RISC. When comparing our data set with *in silico* predicted and conserved miR-124 targets (TargetScan) we found that around 10% of the genes overlap. This proportion is similar to other experimental efforts to identify miRNA targets^38^ and highlight the need for experimental identification of miRNA target networks. It is worth noting that several well-defined miR-124 targets such as PTBP1 and Sox9^39^ were not identified in our analysis since these genes are very lowly expressed in the adult hippocampus. Thus cellular context is very important when establishing the functional role of different miRNAs. Our GO analysis shows that miR-124 controls genes related to transcriptional control and metabolic processes in hippocampal neurons.

We also extended our analysis to include neuron-specific identification of miR-125-targets. miR-125 is highly expressed in neurons, however in contrast to miR-124, also present in glia^30^ where distinct genes are likely to be targeted, making bioinformatical approaches to identify miR-125 targets complicated. Previous studies have suggested that miR-125 regulates genes related to synaptic plasticity and dendritic arborisation^30^,^32^. This is supported by our current analysis where axon guidance was the most enriched GO-term. In addition, our data support a role for miR-125 in the control of protein degradation and cell death. The cellular networks controlled by miR-124 and miR-125 are largely separate, yet both networks indicate important functions for these miRNAs in the adult mouse hippocampus. Thus, the disruption of those networks could lead to severe consequences. In line with this it is interesting to note that dysregulation of both miR-124 and miR-125 have been implicated in Alzheimer´s disease^40^,^41^.

We also found that genes exhibiting lowered level in the RISC due to the competition with miR124sp and miR125sp transcripts, also displayed increased total mRNA levels. This shows that miRNAs at least partially act on their targets by inducing mRNA degradation in neurons, although our data does not exclude that miRNAs also act via translational inhibition . However, it is worth noting that inhibition of miR-124 or miR-125 did not affect AGO2-binding of several validated miR-124 and miR-125 targets (Supplementary Fig. 3). This finding could possibly reflect the known redundancy among miRNAs; that many mRNAs are regulated by more than one miRNA and thereby limits the actual impact of individual miRNA family deficiency on the AGO2 binding of mRNAs. With this mind, it is worth noting that most studies validating miRNA targets use overexpression approaches, which do not allow for the detection of such an effect. These suggestions are also in line with a previous study by Tan *et al.* where only 15% of RISC-associated miR-128 target genes, identified by HITS-CLIP, were upregulated after miR-128 deletion 11. However, a potential drawback of the RIP-seq approach is that it provides a snapshot of miRNA-mRNA interactions (as does HITS-CLIP and related techniques) and therefore cannot take temporal differences in mRNA degradation into account. It is therefore possible that some targets with particularly good binding could be more rapidly degraded and would therefore be less enriched in our analysis. Future studies that attempt to link transcription, miRNA-regulation, total mRNA levels and translation in neurons will be very interesting.

In the current study, we used a neuron-specific promoter to analyse miRNA targets in hippocampal neurons. Given the versatility of AAV vectors, this approach could easily be adopted to other cell types using other promoters or cre/loxP technology^42^. This system could also be used to investigate changes in miRNA regulation in animal models of psychiatric and neurodegenerative disorders. While previous studies in disease models have mainly analysed the expression profile of miRNAs and their mRNA targets in bulk tissue samples of the brain, this approach allows for profiling of miRNA targets from discrete cell populations.

In summary, we have established a novel neuron-specific AGO2-RIP-seq technique that allowed us to quantitatively analyse targets of miRNAs currently bound and processed in the RISC of hippocampal neurons. Our results enhance the understanding of miRNA function in neurons and offer a new technology that allows for straightforward profiling of miRNA targets. In the future, this type of studies is likely to increase our understanding of the role of miRNAs in both the healthy and diseased brain.

## Methods

### AAV vectors

The viral vectors used in this study were pseudotyped AAV2/5 vectors, where the transgene of interest is flanked by inverted terminal repeats (ITR) of the AAV2 packaged in an AAV5 capsid. The transfer plasmids were cloned using standard techniques. The GFP-AGO2 sequences for all vectors used in this work were designed according to^34^, synthesised (Genscript) and cloned into AAV transfer vectors. Transgene expression was driven by a human synapsin promoter and all vectors contained a WPRE-element and a late SV40 poly-A sequence. The sponge sequences contained 8 imperfectly complementary binding sites for miR-292 (ACACTCAAAACCCACGGCACTT),miR-124(TTAAGGCACGTATGAATGCCA) and miR-125 (TCACAAGTTTAGTCTCAGGGA).

The AAV vectors were produced using a double-transfection method with the appropriate transfer plasmid and the helper plasmid containing the essential adenoviral packaging genes, as described previously^43^. Vectors were purified by iodixanol step gradients and Sepharose Q column chromatography. The purified viral vector suspension was titrated with TaqMan quantitative PCR and primers targeting the WPRE sequence. The final titers of the injected AAV were in the range of 2E + 14 – 3E + 14 genome copies/ml.

### AAV vector injections

All animal-related procedures were approved by and conducted in accordance with the committee for use of laboratory animals at Lund University. All mice used in this study were anaesthetised using 2% v/v isofluorane in 1 l/min O_2_. Buprenorphine (Temgesic) was applied subcutaneously in adult C57BL/6 male mice, which subsequently received hippocampal injections of AAV vector suspensions. To this end, four holes were drilled in the scull (two per hemisphere) with a total of six 1 μl deposits. The injection coordinates were: (#1 and #2) AP: −2.0 mm; ML: +/−1.4 mm; DV (from dura): −1.5 mm, (#3 and #4) AP: −2.8 mm; ML: +/−2.7 mm; DV: −1.6 mm and (#5 and #6) AP: −2.8 mm; ML: +/−2.7 mm; DV: −3.7 mm. All vector injections were conducted using a pulled glass capillary (outer diameter 60–80 μm) that was mounted on a 22-gauge needle and attached to a 5 μl Hamilton syringe. The injection rate was 0.4 μl/min and the needle was kept in place for 3 minutes after each injection before it was slowly retracted.

### Immunohistochemistry

For immunohistochemical analysis, mice were transcardially perfused with 4% paraformaldehyde (Sigma), the brains were post-fixed for two hours and transferred to 30% w/v sucrose in water. Brains were sectioned coronally on a microtome (35 μm) and put temporarily in KPBS or stored in an antifreeze solution. Standard immunohistochemistry was applied to free-floating sections, as published in detail elsewhere^44^,^45^. Primary antibodies were diluted as follows: chicken anti-GFP 1:1000 (Abcam), mouse anti-NeuN 1:1000 (Millipore). The dilution factor of the secondary antibodies was 1:500 (Molecular Probes). All sections were counterstained with 4′,6-diamidino-2-phenylindole (DAPI, Sigma-Aldrich, 1:1000).

### FISH and miR.T-GFP transgenic mice

Fluorescence *in situ* hybridisation (FISH) was conducted as previously described^13^ using the miRCURY LNA^TM^ detection probes for miR-124 and miR-125 (5′-DIG and 3′-DIG-labeled; Exiqon). miR.T-GFP transgenic mice were generated, sacrificed, the brain tissue freshly frozen, sectioned and stained as previously described^13^ using standard immunohistochemistry.

### RNA-interacting protein immunoprecipitation

AAV vector-injected mice were decapitated, and the hippocampi were quickly dissected and homogenised (within 20 min) in ice-cold lysis buffer (10 mM HEPES (pH 7.3), 100 mM KCl, 0.5% NP40, 5 mM MgCl2, 0.5 mM dithiothreitol, protease inhibitors, recombinant RNase inhibitors, 1 mM PMSF) using TissueLyser LT (50 Hz, 2 min). Homogenates were centrifuged for 15 min at 16200 × *g*, 4 °C to clear the lysate and a 50 μl sample was saved on ice as INPUT sample. The remaining RNA immunoprecipitation sample (RIP sample) was incubated with anti-GFP-coated Dynabeads® Protein G beads (Life Technologies) at 4 °C for 24 h with end-over-end rotation. The beads were coated with rabbit anti-GFP polyclonal antibody ab290 and mAb6556 (Abcam). After incubation, beads were collected on a Dynamagnet (1 min, on ice) and gently resuspended in low-salt NT2 buffer (50 mM Tris-HCL (pH 7.5), 1 mM MgCl2, 150 mM NaCl, 0.5% NP40, 0.5 mM dithiothreitol, 1 mM PMSF, protease inhibitors and recombinant RNAse inhibitors). The beads were transferred into a new collection tube and washed once with low-salt NT2 buffer, followed by two washes with high-salt NT2 buffer (50 mM Tris-HCl (pH 7.5), 1 mM MgCl_2_, 600 mM NaCl, 0.5% NP40, 0.5 mM DTT, protease inhibitors, 1 mM PMSF and recombinant RNAse inhibitors). After the last washing step, the RNA fraction was resuspended in QIAzol buffer and RNA was isolated from RIP and INPUT samples according to the miRNeasy micro kit (Qiagen).

### RNA extraction and LNA-Quantitative real-time PCR

For entire hippocampal RNA, the hippocampi were quickly dissected from decapitated vector-injected mice, frozen on dry ice and homogenised in TissueLyserLT (50 Hz, 2 min). Total RNA was extracted using the RNeasy mini kit (Qiagen). To synthesise cDNA from miRNA, we used the Universal cDNA synthesis kit (Exiqon) according to the supplier’s recommendations. LNA^TM^ PCR primer sets, hsa-miR-103a-3p, hsa-miR-21-5p and hsa-miR-124-3p were purchased from Exiqon. cDNA from mRNA was synthesised using the Maxima First Strand Synthesis Kit for RT-PCR (Fermentas) according to the supplier’s recommendations. Standard procedures for LightCycler 480 SYBR Green I Master (Roche) qRT-PCR was performed and the data was quantified using the ΔΔCt-method, as previously described^45^. Primers were designed using Primer3 software (http://frodo.wi.mit.edu).

### Analysis of RIP samples

A total of 34 mice were used for RIP analysis. For the RIP validation experiments, we used sham-injected (n = 4) and AAV5-GFP-AGO2 injected (n = 4) mice. For the miR-124sp-RIP validation experiments, we used AAV5-GFP-AGO2.miR292sp (n = 5) and AAV5-GFP-AGO2.miR124sp (n = 5) injected mice. RIP validation was performed as individual biological replicates.

For the sequencing experiments, we used sham-injected (n = 4), AAV5-GFP-AGO2.miR292sp-injected (n = 4), AAV5-GFP-AGO2.miR124sp injected (n = 4), AAV5-GFP-AGO2.miR125sp injected (n = 4) mice. For each RIP-seq samples we pooled the lysate from 2 injected hippocampi (same vector type) in order to limit variation due to differences in vector injection. We also performed a unique biological replicate for each RIP-seq samples in order to confirm the robustness of our approach. We found very small differences between replicates.

cDNA libraries of RIP samples were prepared using the NuGEN Ovation RNA-Seq System including poly-A enrichment. Illumina high-throughput sequencing was applied to the samples (total number of reads: 153 545 538). The 50 bp single end reads were mapped to the mouse genome (mm9) and visualised in USCS genome browser. For the RIP-seq experiments, reads were quantified in all known 3′UTRs (miRbase) of the mouse genome and then used for the conduction of further analysis.

We calculated the relative enrichment of reads in the 3′UTR by dividing the number of reads in GFP-AGO2 RIP samples by the number of reads in sham-injected samples for each 3′UTR. We used sham-injected samples for normalisation since these samples combine a background level of gene expression with the same sample preparation procedure as for GFP-AGO2 RIP-samples. Genes were sorted according to their relative enrichment in GFP-AGO2 samples. From this list, GAPDH, and 50 genes above, and 50 genes below GAPDH were selected for normalisation. We normalised reads to the average read numbers of these 101 selected genes. Genes with read numbers lower than 10 in any sample, were excluded from the analysis. To classify miRNA targets, we used a four-fold enrichment as cut-off when comparing mRNA reads in RIP samples from GFP-AGO2-injected mice to sham-injected mice. This stringent cut-off was used to minimise the potential inclusion of false positives in the downstream analysis. Data is presented as the mean value of two independent biological replicates.

For analysing the effect of the injection of AAV5-GFP-AGO2.miR124sp and AAV5-GFP-AGO2.miR125sp, we ranked all genes in the GFP-AGO2.miR292sp data based on their relative enrichment in AGO2 binding. The gene with the lowest relative enrichment in AGO2 binding was ranked #1, the second lowest #2 and so on. We continued this ranking throughout the list of more than 8000 genes in our data set. Subsequently, we also assigned ranks to the GFP-AGO2.miR-124sp and GFP-AGO2.miR-125sp samples. The ranks of GFP-AGO2.miR292sp were used to produce cumulative graphs (x-axis) comparing the presence of a computationally predicted and evolutionary conserved target site for five separate miRNAs (miR-124, miR-125, let-7, miR-21, miR-292). If the genes with target sites for a miRNA are randomly distributed throughout the ranked list of genes with increasing AGO2 binding, this graph will have a linear appearance. On the other hand, the genes with target sites for a miRNA that is functional in the cell of interest, will have a higher AGO2 binding and will thus be present at the right-most part of the graph, giving this a curved appearance.

To identify genes with the highest reduction in AGO2 binding after sponge injection, we calculated the increase in rank number for each gene in GFP-AGO2 samples compared to miR-sponge samples (for both miR124sp and miR125sp). We focused our analysis on genes present in the top 2177 (>4 fold change in GFP-AGO2 samples) to avoid the inclusion of changes due to noise.

### mRNA sequencing

We used a total of 12 mice for mRNA-sequencing. For the miR-124 experiment, we used 3 mice injected with AAV5-GFP.miR124sp and 3 mice injected with AAV5-GFP. For the miR-125 experiment, we used 3 mice injected with AAV5-GFP.miR125sp and 3 mice injected with AAV5-GFP. cDNA libraries of mRNA samples were prepared using the NuGEN Ovation RNA-Seq System including poly-A enrichment. Illumina high-throughput sequencing was applied to the samples (total read number 456 123 958). The 50 bp single end reads were mapped to the mouse genome (mm9) and visualised in USCS genome browser. Reads were quantified to Refseq. Differentially expressed genes were calculated using the Bioconducter/R package DESeq^46^. Data is presented as the mean value of three independent biological replicates.

### Gene ontology analysis

Gene ontology analysis was conducted using the DAVID bioinformatics database (http://david.abcc.ncifcrf.gov). Genes with a read number >10 from the mRNAseq data of AAV5-GFP control mice, were used as background list for functional annotation analysis (11878 genes). Using medium default stringency, we identified enriched (p < 0.05) Gene Ontology (GO) biological processes (BP) and Kyoto Encyclopedia of Genes and Genomes (KEGG) pathways in a functional annotation chart. KEGG pathways were labelled in bold writing.

### miRNA target site predictions

For the identification of predicted miRNA targets, mouse TargetScan 6.2 was used (http://www.targetscan.org/mmu_61/).

### Statistical analysis

Unpaired parametric t-tests were used to analyse means of two groups. Kolmogorov-Smirnov Z tests were used to determine significance in graphs analysing cumulative fraction of genes vs the fold change after miR-sponge inhibition. The criterion for significance for all analyses was p < 0.05. All data were expressed as mean ± SEM.

## Additional Information

**How to cite this article**: Malmevik, J. *et al.* Identification of the miRNA targetome in hippocampal neurons using RIP-seq. *Sci. Rep.* **5**, 12609; doi: 10.1038/srep12609 (2015).

## Supplementary Material

Supplementary Information

Supplementary Information
