# Supplementary material for: Identification of the miRNA targetome in hippocampal neurons using RIP-seq
Source: Sci Rep. 2015 Jul 28;5:12609. doi: 10.1038/srep12609 (PMC4517465; doi:10.1038/srep12609)
Supplement: Supplementary Information [file srep12609-s1.pdf]

Identification of the miRNA targetome in hippocampal neurons using RIP-seq

Josephine Malmevik[1,2], Rebecca Petri[1,2], Thies Klussendorf[1], Pina Knauff[1], Malin Åkerblom[1], Jenny Johansson[1], Shamit Soneji[1] & Johan Jakobsson[1,3]

Supplementary Figure S1: Validation of the neuron specificity of AGO2-RIPseq. Related to Fig. 2

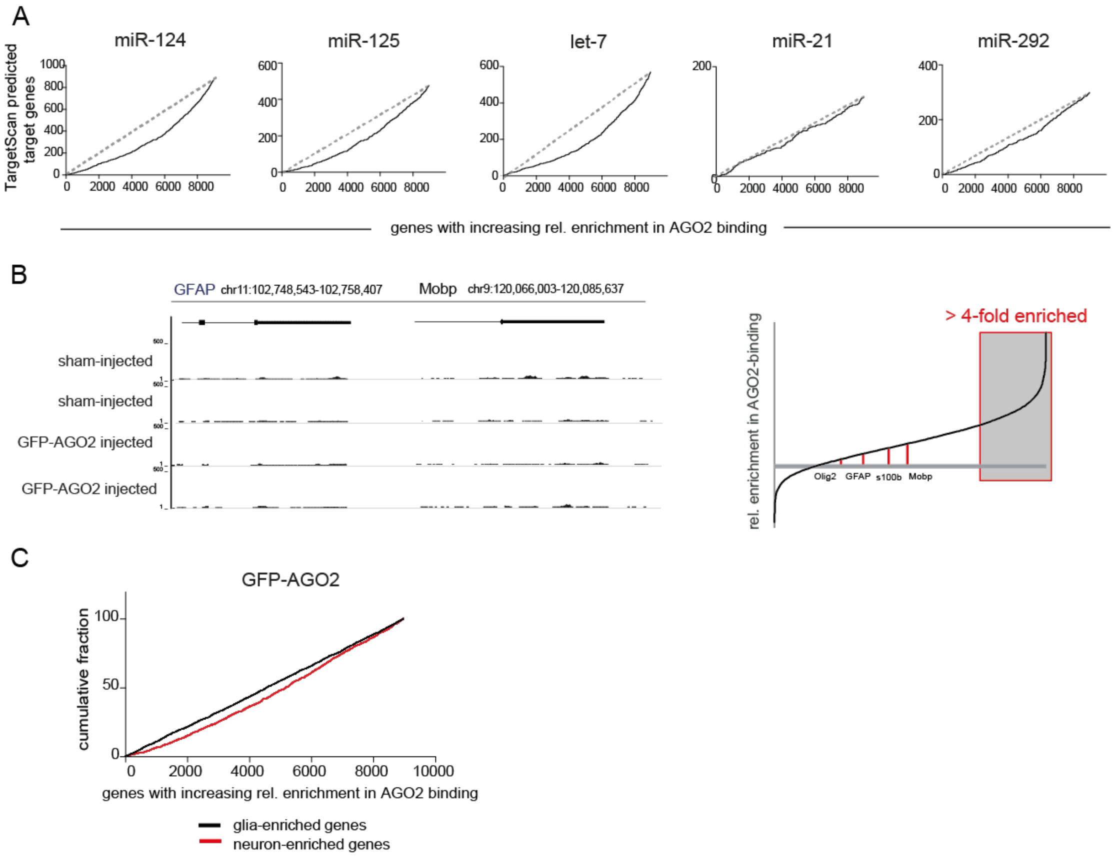

Supplementary Figure S1. (A) Genes with predicted miRNA target sites were cumulatively plotted vs. increasing enrichment in AGO2 binding. Curves extending below the diagonal dotted line indicate that the predicted targets are enriched in the top AGO2-binding genes. (B) Glia-expressed genes (shown here: GFAP and Mobp) showed only negligible reads in their 3'UTR in RIP-seq samples (left panel) and only very minor AGO2 binding (shown here for: Mobp, GFAP, s100b and Olig2, depicted in red, right panel). (C) Genes with increasing AGO2 binding were plotted vs. cumulatively plotted glia-expressed (black line) and neuron-expressed (red line) genes [1]. In contrast to glia, neuron-expressed genes were enriched in AGO2-bound transcripts.

Supplementary Figure S2: Expression of GFP-AGO2-miRsponges in mouse hippocampus. Related to Fig. 4

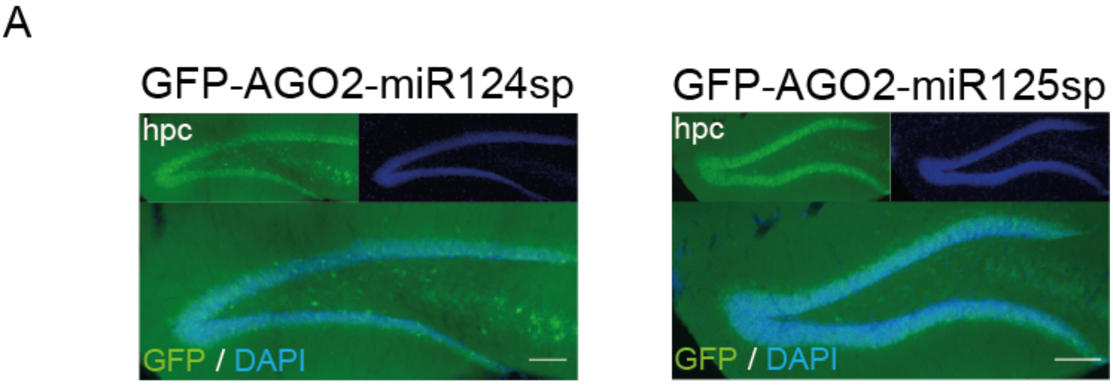

Supplementary Figure S2. (A) Expression of GFP-AGO2-miR-124sp (left panel) and GFP-AGO2-miR-125sp (right panel) in the mouse hippocampus. Scale bars:100 µm.

Supplementary Figure S3: Limited changes in the 500 genes with highest relative enrichment in AGO2 binding after miRNA inhibition.

A

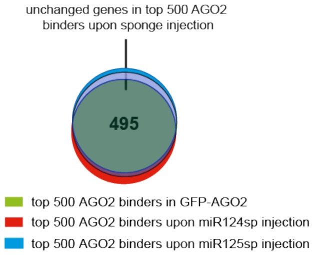

B

gene name	rank among 8971 genes in GFP-AGO2	rank among 8971 genes in GFP-AGO2.125sp	rank among 8971 genes in GFP-AGO2.124sp	predicted conserved miRNA target sites (TargetScan)
Fam78a	8971	8971	8971	miR-27abc/27a-3p, miR-137/137ab, miR-182, miR-128/128ab, miR-101/101ab, miR-218/218a, **miR-124/124ab/506**, **miR-125a-5p/125b-5p/351/670/4319**, miR-96/507/1271, miR-335/335-5p
Frat2	8970	8969	8951	miR-22/22-3p,miR-29abcd,miR-26ab/1297/4465, miR-31, miR-23abc/23b-3p
Ddit4	8969	8968	8961	miR-22/22-3p, miR-221/222/222ab/1928, miR-153, miR-223, miR-7/7ab, miR-101/101ab, miR-30abcdef/30abe-5p/384-5p, miR-181abcd/4262, miR-25/32/92ac/ 363/363-3p/367, miR-200bc/429/548a
Sec24d	8968	8963	8962	miR-204/204b/211
Zswim5	8967	8959	8964	**miR-125a-5p/125b-5p/351/670/4319**, let-7/98/4458/4500, miR-326/330/330-5p, miR-202-3p
Dhx8	8966	8957	8963	No predicted miRNA target sites
Scrt2	8965	8770	8767	miR-27abc/27a-3p, miR-17/17-5p/20ab/20b, 5p/93/106ab/427/518a-3p/519d,miR-33ab/335p, miR-128/128ab
Zbtb34	8964	8965	8969	miR-15abc/16/16abc/195/322/424/497/1907, miR-448/448-3p, miR-29abcd, miR-374ab, miR-139-5p, miR-224, miR-23abc/23b-3p, miR-290-5p/292-5p/371-5p/293, miR-103a/107/107ab, miR-410/344de/344b-1-3p, miR-31, miR-340-5p, miR-101/101ab, miR-141/200a, miR425/425-5p/489, miR-125a-5p/125b-5p/351/670/4319, miR-153, miR-181acd/4262, miR-25/32/92abc/363/363-3p/367, miR-27abc/27a-3p, miR-192/215, miR-144, miR-137/137ab, miR-132/212/212-3p, miR-135ab/135a-5p,miR-9/9ab, miR-204/204b/211
Slc44a5	8963	8964	8960	miR-9/9ab, miR-135ab/135a-5p, **miR-124/124ab/506**, miR-28-5p/708/1407/1653/3139
Sema7I	8962	8952	8957	miR-27abc/27a-3p, miR-17/17-5p/20ab/20b, 5p/93/106ab/427/518a-3p/519d,miR-33ab/335p, miR-128/128ab

C

miR-124 validated target genes

gene name	rank among 8971 genes in GFP-AGO2	rank among 8971 genes in GFP-AGO2.125sp	rank among 8971 genes in GFP-AGO2.124sp
Itgb1	8961	8967	8968
Insm1	8954	8940	8948
Btg2	8950	8928	8941
Dll1	8924	8685	8708
Ccne1	8910	8713	8881
Mbp	8843	8822	8791
Adipor2	8803	8828	8824
Dlk1	8783	8493	8120
Dhcr24	8773	8508	8477
Socs2	8769	8882	8866

miR-125 validated target genes

gene name	rank among 8971 genes in GFP-AGO2	rank among 8971 genes in GFP-AGO2.125sp	rank among 8971 genes in GFP-AGO2.124sp
Klf13	8253	8071	7756
Igfbp3	8000	7214	6835
Rabl6	7696	7227	7125
Rhebl1	7580	8002	7525
Ddx19b	7542	6843	7557

Supplementary Figure S3. (A) Out of the 500 genes with highest relative enrichment in AGO2 binding, only a low number of miR-124 and miR-125 target genes were removed from the RISC after inhibition of each respective miRNA. (B) Top ten genes showing the highest relative enrichment in AGO2 binding. (C-D) Set of validated miR-124 target genes (C) and miR-125 target genes (D) among the 500 genes with the highest enrichment in AGO2 binding.

Table S1. All mapped genes sorted according to their enrichment in AGO2 binding. Table S1 is found in a separate Excel sheet. Related to Fig. 2.

Table S2. 300 genes with most reduced AGO2 binding after miR124sp injection. Table S2 is found in a separate Excel sheet. Related to Fig. 4.

Table S3. 300 genes with most reduced AGO2 binding after miR125sp injection. Table S3 is found in a separate Excel sheet. Related to Fig. 5
